# Contrast of 83% in reflection measurements on a single quantum dot

**DOI:** 10.1038/s41598-019-45259-z

**Published:** 2019-06-19

**Authors:** Pia Lochner, Annika Kurzmann, Rüdiger Schott, Andreas D. Wieck, Arne Ludwig, Axel Lorke, Martin Geller

**Affiliations:** 1 University of Duisburg-Essen, Faculty of Physics and CENIDE, D-47057 Duisburg, Germany; 2 Ruhr-Universität Bochum, Lehrstuhl für Angewandte Festkörperphysik, D-44780 Bochum, Germany; 3 Solid State Physics Laboratory, ETH Zurich, 8093 Zurich, Switzerland

**Keywords:** Quantum dots, Single photons and quantum effects

## Abstract

We report on a high optical contrast between the photon emission from a single self-assembled quantum dot (QD) and the back-scattered excitation laser light. In an optimized semiconductor heterostructure with an epitaxially grown gate, an optically-matched layer structure and a distributed Bragg reflector, a record value of 83% is obtained; with tilted laser excitation even 885%. This enables measurements on a single dot without lock-in technique or suppression of the laser background by cross-polarization. These findings open up the possibility to perform simultaneously time-resolved and polarization-dependent resonant optical spectroscopy on a single quantum dot.

## Introduction

Excitons in epitaxially grown self-assembled semiconductor quantum dots (QDs) are promising candidates for the realization of quantum information and communication technologies^1,2^. Resonance fluorescence is a widely used possibility to address resonantly the exciton transition in a single QD^3–5^ and generate single, indistinguishable photons^6–8^. Commonly, this requires the suppression of the laser background by several orders of magnitude, e.g., by a dark-field technique based on cross-polarization^3,9,10^ or perpendicular QD excitation and photon detection^11,12^. Another possibility is differential reflection (transmission), where the quantum dot is periodically driven in and out of resonance and the QD photons are discriminated from the reflected (transmitted) laser light by a lock-in technique^13,14^. However, to date, a maximum change in reflection (i. e. the contrast as ratio between QD photons and the incident back-scattered laser light) of about 12% was observed^15^, even though a reflectivity of 85% of the incident light by a single point-like dipole is theoretically predicted^16^.

We report here on an optimized semiconductor heterostructure with an embedded self-assembled InAs/GaAs quantum dot that shows record values for the contrast in reflection measurements. The sample consists of an epitaxially grown p-doped gate, a distributed Bragg reflector (DBR) and a layer structure with an optical standing wave that matches the QD layer. This optimized sample structure shows a contrast of up to 83% in vertical geometry, where the optical path of the excitation laser is parallel to the detection way of the QD photons. An even higher value of more than 800% can be obtained by tilting the excitation laser with respect to the detection path. This allows to separate the QD signal from the laser background without the need of cross-polarization or lock-in techniques and enables polarization- and time-resolved optical spectroscopy on a single quantum dot.

## Sample Design

**Figure 1:**
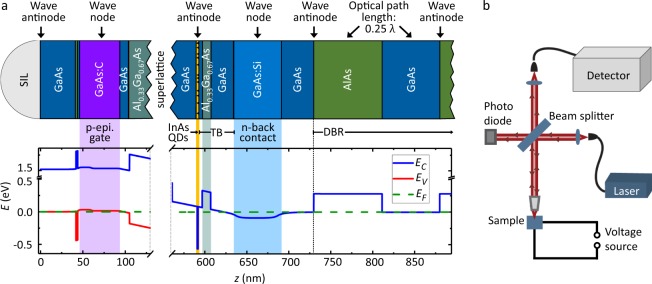
Sample design and measurement setup. (**a**) Schematic sample structure (top) and corresponding band structure (blue: conduction band (*E*_*C*_), red: valence band (*E*_*V*_), green: Fermi level (*E*_*F*_)), calculated with a 1D Poisson solver^31^ (bottom): The InAs QD layer (orange) is separated from the n-doped back contact (bright blue) by a 45 nm thick tunneling barrier TB. The p-doped layer (purple) works as an epitaxial gate, while a 16-fold distributed Bragg reflector (DBR) in the back of the sample provides a high reflectivity in a specific energy range. For an incoming wavelength of 950 nm, the gate is located in a wave node, whereas the QD layer and the beginning of the DBR are positioned in a wave antinode. The solid immersion lens (SIL) on top of the sample increases the collection efficiency. (**b**) Measurement setup: A 10/90 beam splitter reflects the laser light down to the sample, which is cooled to 4.2 K in a bath cryostat. There, the light is focused to a 1 *μ*m diameter spot and addresses a single QD. The QD- and reflected laser photons are then coupled into a fiber and guided to the detection path.

A usual molecular beam epitaxy (MBE) grown sample for resonant optical measurements contains a layer of self-assembled QDs in a diode-like (Al)GaAs heterostructure, where the dots are separated from a n-doped back contact by tunneling barrier^4^. In the sample presented here and shown schematically in Fig. 1a, the gate is an epitaxially grown p-doped layer. In comparison to a commonly used gold gate on the surface, it is transparent for the laser and QD light and scatters less photons due to its single crystalline structure and smoothness^17,18^. Additionally, a 16-fold distributed Bragg reflector (DBR) is included at the bottom of the sample underneath the back contact in order to increase the reflectivity. Given that the large difference of the refractive indices at the sample surface will create an antinode, the QDs are placed in an antinode of the standing wave, while the epitaxial gate and the back contact are placed in a wave node. To achieve this, the structure was designed regarding the optical path length *OPL* = *nd* with the refractive index *n* and the layer thickness *d*. A typical QD excitonic recombination wavelength of 950 nm was assumed for this design. The QDs positioned in an antinode leads to a better coupling of the QD excitonic transitions to the laser light field. The center of the p-doped layer of the epitaxial gate and the n-doped layer of the back contact are both positioned in a wave node, as this decreases the light absorption in a layer that contains a large number of free charge carriers. For this purpose, the epitaxial gate is covered by GaAs and AlAs with an overall optical path length of approximately 0.16*λ*. To increase the collection efficiency, a solid immersion lens (SIL) consisting of zirconia is positioned on top of the structure. Altogether, this yields the sample structure (see methods for more details) and the corresponding band structure visualized in Fig. 1a.

## Resonance Fluorescence Measurements with Polarization Suppression

**Figure 2:**
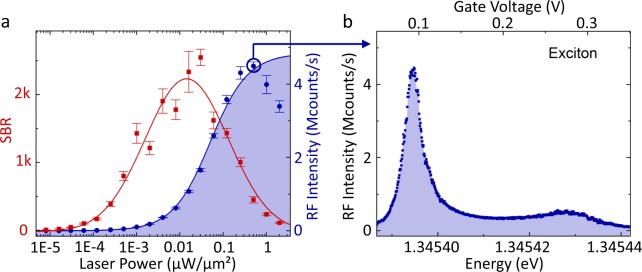
Resonance fluorescence (RF) measurements. (**a**) The intensity of the RF signal of the exciton (blue dots) and the QD signal to total background ratio (SBR, red rectangles) are plotted for increasing laser excitation power. The intensity is the sum of the maximum count rates of both fine-structure split exciton resonances for each laser power. The fit (blue line) excludes data points for laser powers above 0.5 *μ*W/*μ*m^2^ because here, the measured intensity is strongly affected by photon-induced electron capture^4^. The red line is just a guide to the eye for the SBR data points. Panel (**b**) shows a single gate voltage scan at fixed laser energy (325.315 THz) of the fine structure split excitonic lines at the highest count rate. All count rates are measured directly on an avalanche photodiode (APD) and corrected by a small background intensity, given by dark counts of the APD and laser photons that are not suppressed by the cross-polarization technique.

The excitonic photon count rate of a QD in the sample was first investigated by resonance fluorescence measurements, where the backscattered laser light was suppressed by the cross-polarization technique (see methods). Figure 2a shows that, with increasing laser power, the resonance fluorescence intensity increases to the highest value of 4.5 Mcounts/s (Fig. 2a). At the highest laser intensity, the resonance fluorescence intensity is decreasing again due to photon-induced electron capture into the QD^4^. The solid line is a fit for a resonantly driven 2-level system^19^. A spectrum of the exciton transition at the maximum count rate, obtained by a gate voltage scan is shown in Fig. 2b. The shown resonance fluorescence intensities of the exciton resonances correspond to the values measured on an avalanche photodiode (APD), only corrected by a small background intensity. We receive the maximum count rate of 4.5 Mcounts/s by summation of both maxima of the fine-structure split exciton resonances. Considering the transmission of the setup and the characteristics of the detector, this can be converted to a count rate at the first lens, which is about 12-fold higher (see Methods). In comparison, in the same setup, a maximum excitonic count rate of 150 kcounts/s was obtained from a previous sample^5^. As a result of the high photon rate, the QD signal to total background ratio (SBR) has a maximum value of more than 2500 at a laser power of 0.03 *μ*W/*μ*m^2^. For high laser excitation power above the saturation point the SBR decreases again^19,20^. These resonance fluorescence measurements demonstrate that the sample design enables a high quantum dot photon emission and collection efficiency.

## Resonance Fluorescence without Cross-Polarization

**Figure 3:**
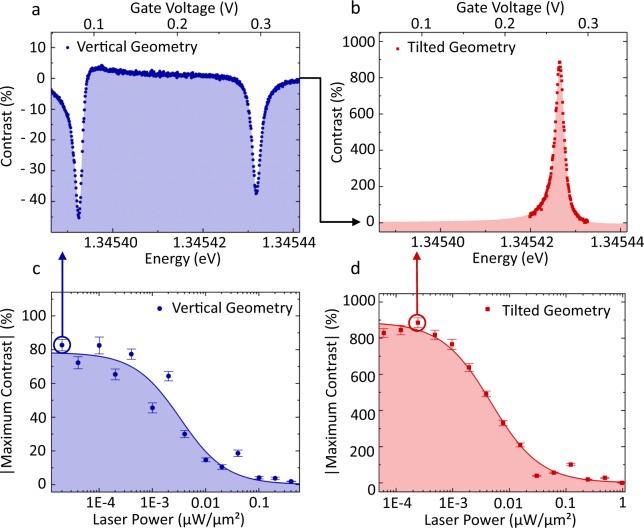
Contrast measurements in reflection without lock-in technique or cross-polarization. Single gate voltage scans of the exciton at the highest contrast are shown in vertical (**a**) and tilted geometry (**b**). The gate voltage was tuned while the laser energy was fixed (325.315 THz in vertical, 325.323 THz in tilted geometry), the corresponding energy was determined by the measured Stark shift. The slightly different excitation energies for titled and vertical geometry do not affect the results. All contrast measurements were obtained without lock-in technique. In vertical geometry, the incident laser light was unpolarized and for detection, a CCD camera in the image plane of a spectrometer was used. Destructive interference is observed due to the phase relation in this alignment. In tilted geometry, the signal was detected by an APD. Only one of the excitonic transitions was observable here due to appropriate linearly polarized incident laser light. Panel (**c**) and (**d**) show the absolute value of the maximum contrast for increasing excitation laser power in vertical and tilted geometry, respectively.

Resonant optical measurements at both exciton transitions without cross-polarization are discussed in the following. The ratio between QD signal Δ*R* and reflected laser signal *R* is called “contrast” (Δ*R*/*R*)^14,21^. Figure 3 shows the measured contrast for an alignment of the excitation beam in vertical and tilted geometry. In vertical geometry, the incoming laser beam is perpendicular to the sample surface. During adjusting the measurement until the maximum contrast in this geometry is reached, the phase between the QD photons and the reflected laser light can change. Here, it leads to destructive interference^21^, so that the contrast has a negative sign (see Fig. 3a). Note, that we can add the contrast from both exciton transitions in Fig. 3a, as the contrast is the ratio between all QD photons and scattered laser intensity. Linear polarization and alignment on one of the exciton transitions would yield the same maximum contrast on a single transition. The measurement of the contrast in vertical geometry was directly done on a Si-CCD camera at the exit of a spectrometer without any need of a lock-in technique. In this geometry, a maximum contrast of 83% is reached (see Fig. 3a). Figure 3c shows the maximum contrast for increasing laser excitation power up to 0.4 *μ*W/*μ*m^2^.

In the other geometry, the laser excitation is tilted by an angle of maximum 0.1° by small changes in the alignment of the excitation path of the microscope head (see Fig. 1b and methods). The disadvantage of this alignment is a decreased photon count rate from the QD but on the other hand, in comparison to the QD photons, the reflected laser photons are only partly collected by the fiber. Thus, the contrast exceeds 800% and reaches a value of 885% at low excitation powers (Fig. 3b,d). This means, that for about eight QD photons only one background photon (consisting of dark counts of the APD and laser photons that are not suppressed by the cross-polarization technique) is measured. Due to the smaller count rates in this measurement, the photons were detected by an APD, again without the cross-polarization technique. Furthermore, the incident laser light was linearly polarized in an orientation that suppresses one of the two fine-structure split excitonic resonances and maximizes the second resonance.

## Simulation

In the following section, the transfer matrix algorithm^22^ will be used to simulate the reflectivity and, hence, the contrast of the sample structure for different device geometries. As all angles between incident light and surface normal are neglected, these simulations are comparable with the measurements in vertical geometry. These simulations in combination with the measurement demonstrate that the main contribution for the observed high contrast has to be an enhanced interaction between the laser light field and the QD dipole transitions.

In the transfer matrix algorithm, for every layer *i* in the sample the transfer matrix

1 $${M}_{i}=(\begin{array}{cc}\cos (k{n}_{i}{d}_{i}) & \frac{1}{{\rm{i}}{{n}}_{i}}\,\sin (k{n}_{i}{d}_{i})\\ -{\rm{i}}{{n}}_{i}\,\sin (k{n}_{i}{d}_{i}) & \cos (k{n}_{i}{d}_{i})\end{array}),$$

is assumed, where *k* is the wave number of the resonant laser in the vacuum, *n*_*i*_ the refractive index and *d*_*i*_ the thickness of layer *i*. To obtain the transfer matrix for the quantum dot, the limit *M*_*i*_(*d*_*i*_ → 0) is calculated and

2 $${M}_{QD}=(\begin{array}{cc}1 & 0\\ \frac{{\sigma }_{2D\mathrm{,1}}}{{\varepsilon }_{0}{c}_{0}} & 1\end{array})+(\begin{array}{cc}1 & 0\\ \frac{{\sigma }_{2D\mathrm{,2}}}{{\varepsilon }_{0}{c}_{0}} & 1\end{array})$$

is obtained. The two-dimensional conductivity *σ*_2*D*,*j*_, where *j* = 1, 2 denotes the two excitonic transitions, is given by

3 $${\sigma }_{2D,j}=\frac{{e}^{2}{n}_{2D}{f}_{j}}{{m}_{0}}\frac{-{\rm{i}}\omega }{{\omega }_{\mathrm{0,}j}^{2}-{\omega }^{2}-{\rm{i}}\omega {\Gamma }_{j}},$$

with the laser frequency *ω*, the resonance frequency of the excitonic transition *ω*_0_ and the dephasing rate Γ^21^. As only one QD per excitation laser spot is present (otherwise, one would see more resonances in the spectra in Figs 2b and 3a,b), the areal density *n*_2*D*_ is defined as $\frac{1}{A}$ (with *A* as area of the laser spot)^21^. *f* usually represents the oscillator strength, but in our simulation it is used as a fit parameter and thus also includes other effects which affect the interaction between the QD dipole transition and the light field, e.g., the Purcell effect. To avoid confusion, in the following instead of *f*, *c* is used for this fit parameter. A detailed analysis of the interaction between the excitation laser field and the QD as two-level system^23,24^ is not part of the simulation here. The calculations should rather show the influence of the DBR and the epitaxial gate on the contrast measurements.

The matrix *M* of the entire structure is the product of the matrices for each layer $M={\prod }_{i}{M}_{i}$. The reflectance *r* of the structure is then given by

4 $$r=\frac{({m}_{11}+{m}_{12}{n}_{e}){n}_{a}-({m}_{21}+{m}_{22}{n}_{e})}{({m}_{11}+{m}_{12}{n}_{e}){n}_{a}+({m}_{21}+{m}_{22}{n}_{e})},$$

where *m*_*kl*_ are the matrix elements of *M* and *n*_*a*_ and *n*_*e*_ are the refractive indices for zirconia above and GaAs underneath the sample structure, respectively^22^. The solid immersion lens on top of the sample has only been taken into account by the refractive index above the sample structure, because its thickness is much bigger than the wavelength of the excitation laser and the QD emission and its surface is not plane-parallel. The square of the absolute value of the reflectance is the expected reflectivity of the sample *R* = |*r*|^2^. The contrast, which is the value that is measured (see Fig. 3), is defined as the quotient of the reflectivity in resonance and the reflectivity out of resonance, i. e. the reflectivity without the QD emission.

## Simulation Results and Discussion

**Figure 4:**
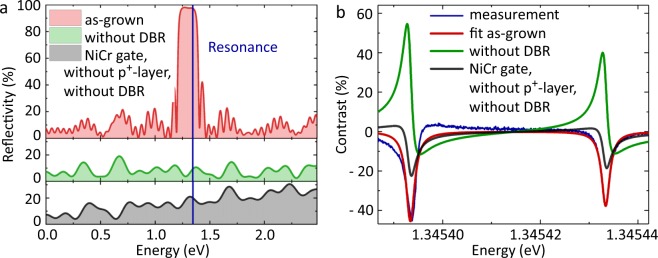
Simulation of the reflectivity and contrast. (**a**) Calculated reflectivity of the sample depending on the photon energy *E*. At the resonance energy of the QD (blue line), the reflectivity of the as-grown sample in this work (red line) is up to 98%. Removing the DBR leads to a decreased reflectivity (green line) at the QD resonance. Replacing in addition the p^+^-doped epitaxial gate by a 7 nm thick NiCr gate on top of the sample (black line) increases the reflectivity slightly again. Panel (**b**) shows the corresponding simulation of the expected contrast. The contrast for the as-grown sample (red line) is a fit to the measurement (blue line), shown in Fig. 3a. Removing the DBR (green line) or the epitaxial gate (black line), respectively, leads to a different line shape in the contrast measurement.

The results of these simulations can be seen in Fig. 4. Three different structures are considered here: (i) The optimized sample design as described in Fig. 1a, (ii) the same sample structure without DBR layers and (iii) a sample structure where the DBR layers are removed and the epitaxial gate is replaced by a 7 nm thick NiCr gate to account for the commonly used evaporated metal gates. As expected, the simulation for the as-grown sample (case (i), red line in Fig. 4a) shows a high reflectivity of 98% at the excitonic resonance energy (blue line), while the reflectivity of the structures without DBR (case (ii) and (iii), green and black line) have a much lower value at the exciton transition energy (Fig. 4a). Interestingly, the structure with metal gate (iii) shows a higher reflectivity than the one with epitaxial gate (ii). This implies that the metal gate reflects a higher amount of the incident laser photons before they can reach the quantum dot.

The red line in Fig. 4b is a fit to the contrast measurement in vertical geometry (blue line) and shows a good agreement for values of *c*_1_ = 311 and *c*_2_ = 240 for the left and the right excitonic transition, respectively. If *c* would only represent the oscillator strength, it was more than one order of magnitude higher than in previous publications^21,25–27^. As the interaction between the QD and the light field is squeezed into *c* as fitting parameter, we can already conclude here, that this interaction seems to be strongly enhanced in our sample structure, showing such a high value for the fit parameter.

This assumption can be supported by the following simulations: The green line in Fig. 4b shows the results for case (ii) without the DBR, where the same fitting parameter values as received for case (i) are used. The contrast has not significantly changed, only a different line shape is observed caused by an altered phase relation between the reflected laser light and the QD photons. That means, the DBR itself has almost no influence on the contrast of a resonant measurement, as the incident laser photons are back-reflected by the DBR equally to the QD photons. A minor effect of the DBR is related to the interaction between QD dipole transitions and light field by the Purcell effect. The Purcell effect enhances this interaction, however, in samples similar to ours only by a negligible small factor of about 1.2^28^. Finally, substituting the epitaxial gate by a NiCr gate in the simulations decreases the contrast by a factor of about 2 when again the same fitting parameter values as received for case (i) are used (see black line in Fig. 4b), however, it is not as strongly decreased as expected from previous reports^15^.

Our simulation show that the major effect comes most likely from the position of the wave node and antinode of the standing wave which builds up due to the DBR in the bottom of the sample structure: (i) The interface of the epitaxial gate is buried inside the sample and the wave node is positioned in the middle of the highly p-doped GaAs, leading to a smaller photon absorption in the area of high charge carrier density. A metal gate on top of the sample would pin the wave node to the surface and reflect part of the incident laser light before it reaches the quantum dots. This has most probably the greatest effect on the contrast. (ii) The position of the QD in a wave antinode enhances the interaction between light field and QD dipole transitions, visible as huge *c* in our simulations.

Independent from the simulations, the epitaxial gate is much smoother and cleaner than a metal gate, which results in less scattering of the laser and QD light^17^.

## Conclusion

In conclusion, we have demonstrated a record value for the contrast in differential reflection measurements of up to 83%. In a tilted geometry of the laser excitation, this value can be even enhanced to above 800%. Simulations using the transfer matrix algorithm show that the main contribution to such a high contrast most likely is in a strong interaction between the laser light field and the QD dipole transitions, visible as huge fitting parameter that takes, i. a., the oscillator strength into account. This leads us to the conclusion, that the buried epitaxial gate within a node of the standing wave which builds up due to the DBR underneath the diode structure of the sample and positioning of the QD in a wave antinode yields a sample with a very high contrast between QD photons and back-scattered laser light. Our results guide the way to further optimization of the sample structure and opens up new possibilities to investigate single self-assembled quantum dots without the need of cross-polarization or phase-sensitive lock-in techniques.

## Methods

### Sample design

The sample used in this work was grown by molecular beam epitaxy (MBE) and has a layer sequence as follows: A 50 nm thick buffer layer of GaAs and a periodic superlattice (SPS) with 20 layers of 2 nm AlAs and 2 nm GaAs were grown to smoothen the substrate surface and capture impurities, followed by the distributed Bragg reflector (DBR). The DBR consists of 16 sequences of 68.6 nm GaAs and 81.45 nm AlAs, having an optical path length of 0.25*λ* at a typical QD wavelength of 950 nm. After 43 nm of GaAs (to position the beginning of the DBR in a wave antinode), a 50 nm thick silicon n-doped GaAs (*N*_*D*_ = 2 · 10^18^ cm^−3^) layer follows as back contact. The following tunneling barrier consists of 30 nm of GaAs, 10 nm of Al_0.33_Ga_0.67_As and 5 nm of GaAs. After that, a layer of self-assembled InAs QDs was grown in the Stranski-Krastanov growth mode, partially capped flushed and covered by 30 nm of GaAs. A SPS consisting of 41 layers of 3 nm AlAs and 1 nm GaAs acts as a current blocking layer, while the following 292 nm of Al_0.33_Ga_0.67_As increase the lever arm of the structure and are fit to an optical length which shifts the QD into a wave antinode. 10 nm of GaAs separate this layer from the epitaxially grown gate, which consists of 30 nm of carbon doped GaAs (*N*_*A*_ = 2 · 10^18^ cm^−3^) and 15 nm highly carbon doped GaAs (*N*_*A*_ = 1 · 10^19^ cm^−3^). Finally, 1 nm GaAs, 2 nm AlAs and 42 nm GaAs are deposited to bury the epitaxial gate and shift it to a wave node.

For the electrical contacts, the n-doped back contact is reached in chemical wet etching to the GaAs layer underneath the p-doped layer and deposition of 10 nm Ni, 60 nm Ge, 120 nm Au, 10 nm Ni and 100 nm Au and alloying. The epitaxial top-gate is accessed by chemical wet etching to the p-doped layer and deposition of 10 nm Au, 15 nm Cr and 200 nm Au. Finally, a zirconia solid immersion lens (SIL) is positioned on top of the gate. The sample structure can be seen in Fig. 1a.

### Experimental methods

A confocal microscope setup is used to address a single self-assembled QD, shown schematically in Fig. 1b. The light from a tunable laser diode is fed into the microscope and reflected towards the sample by a 10/90 beam splitter. The sample is cooled to 4.2 K in a liquid He bath cryostat. An objective lens with a *NA* of 0.68 focuses the laser to a spot size of about 1 *μ*m, giving a laser spot area *A* of 0.78 *μ*m^2^. Together with the SIL, the objective lens yields a collection efficiency of 5.8%^29^. The resonant laser excitation and QD photon detection is aligned along the same path with a microscope head that contains two 10/90 beam splitter and two polarizers. Cross-polarization enables a suppression of the spurious laser scattering into the detection path by a factor of more than 10^7^. Due to the cross-polarization in this method, the incident laser light is linearly polarized, which can favor the excitation of one of the two fine-structure split exciton resonances. The perpendicular linear polarizer in the detection arm suppresses (apart from the reflected laser light) also some of the QD photons before they reach the detector, which again can change the relative peak intensities.

The losses of QD photons in this setup comprise the losses in the quartz glass window of the sample tube (0.02), in the two beam splitters (0.1 each), in the linear polarizer in the detection arm (0.5) and in the coupler to the detection fiber (0.3). The objective is anti-reflection coated, so the losses here are negligible. Additionally, for the used wavelength, the APD has an efficiency of 0.35. At the highest count rate, a correction factor of 1.15 due to detector dead times has to be taken into account. Altogether, the measured count rates are about 12-fold smaller than the rates at the first objective lens.

The gate and the back contact of the sample can be connected to a voltage source to tune the resonance frequency of the QD state by the Stark effect^30^. To measure a small contrast in differential reflection, i.e. without suppressing the laser background by cross-polarization, a square pulse can be applied to the gate and the signal can be detected phase sensitive by a photodiode connected to a lock-in amplifier^13^.

### Simulation

In the transfer matrix algorithm one receives the transfer matrix for the quantum dot in Equation 2 calculating the limit *M*_*i*_(*d*_*i*_ → 0). In detail, the different entries can be calculated like

5 $${M}_{QD\mathrm{,11}}={M}_{QD\mathrm{,22}}=\mathop{\mathrm{lim}}\limits_{{z}_{i}\to 0}\,\cos (knz)\mathrm{=1}$$

6 $${M}_{QD\mathrm{,12}}=\mathop{\mathrm{lim}}\limits_{z\to 0}\frac{1}{{\rm{i}}n}\,\sin (knz)\approx \mathop{\mathrm{lim}}\limits_{z\to 0}\frac{1}{{\rm{i}}n}(knz-\frac{{(knz)}^{3}}{6})=\mathop{\mathrm{lim}}\limits_{z\to 0}\frac{1}{{\rm{i}}}(kz-\frac{{k}^{3}{n}^{2}{z}^{3}}{6})\mathrm{=0}$$

7 $${M}_{QD\mathrm{,21}}=\mathop{\mathrm{lim}}\limits_{z\to 0}-{\rm{i}}n\,\sin (knz)\approx \mathop{\mathrm{lim}}\limits_{z\to 0}-{\rm{i}}n(knz-\frac{{(knz)}^{3}}{6})=\mathop{\mathrm{lim}}\limits_{z\to 0}-{\rm{i}}(k{n}^{2}z-\frac{{k}^{3}{n}^{4}{z}^{3}}{6})=-\,{\rm{i}}k{n}^{2}z=\frac{{\sigma }_{2D}}{{\varepsilon }_{0}{c}_{0}},$$

with $${n}^{2}=\frac{{\rm{i}}\sigma }{\omega {\varepsilon }_{0}}$$ and *σ*_2*D*_ = *σ*_3*D*_*z*.

## Data Availability

The data that support the findings of this study are available from the corresponding author on request.
