# Author Correction: Contrast of 83% in reflection measurements on a single quantum dot

**DOI:** 10.1038/s41598-019-54298-5

**Published:** 2019-11-25

**Authors:** Pia Lochner, Annika Kurzmann, Rüdiger Schott, Andreas D. Wieck, Arne Ludwig, Axel Lorke, Martin Geller

**Affiliations:** 1 University of Duisburg-Essen, Faculty of Physics and CENIDE, D-47057 Duisburg, Germany; 2 Ruhr-Universität Bochum, Lehrstuhl für Angewandte Festkörperphysik, D-44780 Bochum, Germany; 3 Solid State Physics Laboratory, ETH Zurich, 8093 Zurich, Switzerland

Correction to: *Scientific Reports* 10.1038/s41598-019-45259-z, published online 19 June 2019

In the original version of this Article, the authors Andreas D. Wieck and Arne Ludwig were incorrectly affiliated with ‘Solid State Physics Laboratory, ETH Zurich, 8093 Zurich, Switzerland’. The correct affiliation is listed below.

Ruhr-Universität Bochum, Lehrstuhl für Angewandte Festkörperphysik, D-44780 Bochum, Germany

This error has now been corrected in the PDF and HTML versions of the Article.

